# A meta-analysis of stroke risk following herpes zoster infection

**DOI:** 10.1186/s12879-017-2278-z

**Published:** 2017-03-07

**Authors:** Fawziah Marra, Jeremy Ruckenstein, Kathryn Richardson

**Affiliations:** 1 University of British Columbia, 2405 Wesbrook Mall, Vancouver, BC V6T 1Z3 Canada; 2 University of East Anglia, Norwich, United Kingdom

**Keywords:** Herpes zoster, Meta-analysis, Stroke, Transient ischemic attacks, TIA, Aging

## Abstract

**Background:**

The incidence of herpes zoster (HZ) is increasing and poses a significant health concern to aging populations. Several studies suggest an increased risk of stroke following zoster infection, but the results are conflicting. We conducted a systematic review and meta-analysis to determine if stroke risk is increased following HZ infection.

**Methods:**

A search of MEDLINE, EMBASE, Google scholar, Web of Science, CAB Direct, Cumulative Index to Nursing and Allied Health Literature, and Evidence Based Medicine Reviews was conducted for observational studies of adults with HZ infection that examined stroke and TIA risk from January 1, 1966 to May 31, 2016. Adjusted relative risks reported for similar follow-up durations were pooled across studies separately using random-effects inverse variance models.

**Results:**

Data were pooled from nine studies. Relative risk for stroke after zoster was 1.78 (95% CI 1.70–1.88) for the first month following herpes zoster, dropping progressively to 1.43 (95% CI 1.38–1.47) after 3 months, to 1.20 (95% CI 1.14–1.26) after 1 year. We found that stroke risk increases by a larger margin during the first month after a herpes zoster ophthalmicus episode: relative risk 2.05 (95% CI 1.82–2.31). The risk remains elevated one year after the acute episode.

**Conclusions:**

Herpes zoster is an established risk factor for increasing the risk of stroke, especially shortly after infection. Vaccination should be encouraged in patients at high risk of cardiovascular disease.

**Electronic supplementary material:**

The online version of this article (doi:10.1186/s12879-017-2278-z) contains supplementary material, which is available to authorized users.

## Background

Varicella zoster virus (VZV) is a member of the herpes virus family with the characteristic capacity to persist in the body after primary infection and cause latent infection with the risk of reactivation [1]. Primary infection with varicella, also known as chickenpox, is a contagious but a relatively harmless disease that typically affects susceptible children, although it can affect naïve adults as a more severe condition [2, 3]. Viremia during primary infection allows VZV to seed the cranial-nerves or dorsal-root ganglia and lie dormant until reactivation [1]. Reactivation and secondary VZV infection typically occurs in older individuals, perhaps due to compromise of the immune system through medical conditions or medications, and results in herpes zoster (HZ) infection, commonly known as shingles [4]. In VZV reactivation, the virus multiplies and migrates along the nerve to the corresponding dermatome. Postherpetic neuralgia, or pain persisting for 90 days or more after rash starts, is the most common complication of HZ [5, 6]. Because of its reactivation within the nervous system, HZ can cause many other neurological complications including ophthalmicus, a life threatening condition which frequently requires hospitalization and antiviral treatment [7, 8]. Ramsay Hunt syndrome, meningitis, encephalitis, and transverse myelitis are also rare neurologic complications of zoster [7, 9, 10]. Recently, investigators have published several reports on patients with zoster being at higher risk for ischemic or hemorrhagic strokes, however some studies do not find long-term associations and disagree over the time period at which zoster patients remain at risk of stroke outcomes [11]. With over 90% of individuals in the United States showing serological evidence of exposure to VZV and because one in 3 individuals will experience zoster over their lifetime, [4] the potential for HZ reactivation to increase the risk of stroke is a relevant health concern in today’s society. The overall objective of our paper was to conduct a systematic review and pool the results of the studies evaluating stroke risk following an acute episode of herpes zoster.

## Methods

This systematic review and meta-analysis was reported according to the MOOSE guidelines for the reporting of observational studies [12].

### Data sources and search strategy

We conducted a search of MEDLINE EMBASE, Google scholar, Web of Science, CAB Direct, Cumulative Index to Nursing and Allied Health Literature (CINAHL), and Evidence Based Medicine Reviews (EBM) for articles reporting on herpes zoster infection and stroke risk from January 1, 1966 to May 31, 2016. EBM is a collection of seven different libraries including the American College of Physicians journal club, Cochrane Central, Cochrane Systematic Reviews, Cochrane Methodology, Database of Abstracts of Reviews of Effectiveness, Health Technology Assessments, and National Health Services Economic Evaluation Database. Search terms as keywords, Mesh terms and subject headings included: *varicella* OR *zoster* OR herpe* OR postherpe* OR *herpes ophthalmicus* OR *VZV* OR shingle* AND *stroke* OR *TIA* OR *transient ischemic attack* OR *brain ischemia* OR *cerebrovascular* or *cerebral ischemia*. After pooling the articles and deleting duplicates, a manual review of titles was conducted screening for relevant topics and keywords. Another final manual review of article abstracts was conducted on shortlisted articles. If the article was a published abstract in a journal, it was captured in our search. The literature search was performed by two authors (JR and FM) and customized for each database with the help of a university librarian. Uncertainty and revisions were resolved by consensus.

### Inclusion and exclusion criteria

We included all English studies which evaluated either stroke or transient ischemic attacks (TIA) in humans 18 years of age or older following an acute episode of herpes zoster or herpes zoster ophthalmicus (HZO). We excluded cases and case series reports, and literature reviews. For published abstracts found, we contacted the authors to obtain the full manuscript, but if unavailable, we excluded the study.

### Data extraction study verification and quality assessment

Data was extracted independently by two authors (FM and JR) using a standardized abstraction form. Discrepancies were resolved through discussion with another author (KR). Data extracted from the studies included the author, date of the study, type of study, inclusion and exclusion criteria, number of patients, HZ definition, follow up period, confounders adjusted for, demographics and study outcome data.

Two authors (FM and JR) independently conducted the quality assessment of the studies in an unblinded fashion using the Newcastle-Ottawa quality assessment scale (Additional file 1) [13]. Points were awarded to observational studies for comparability if they controlled or adjusted for age, sex, and cardiovascular disease, such as hypercholesterolemia, hypertension, diabetes, coronary heart disease, atrial fibrillation, peripheral vascular disease, carotid stenosis, valvular heart disease, heart failure, chronic rheumatic heart disease, as these are considered important risk factors for stroke. Self-controlled case-series studies were scored with the cohort studies, however we interpreted ‘cohort representativeness’ as how representative the selected cases were of patients with stroke in the community. Discrepancies were resolved through discussion with another author (KR).

### Statistical analysis

As risk of stroke varied with time since herpes zoster infection we pooled adjusted relative risks (RR) reported for similar follow-up durations (e.g. first 1 month, 3 months, 1 year and beyond 1 year since herpes zoster infection, where possible) across studies separately using random-effects inverse variance models. We also performed subgroup analyses by patients older and younger than approximately 40 years where possible. We chose this cut-off as most studies reported stroke risk stratified by age less than and greater than 40 years. Unfortunately, there were not enough studies reporting results in the over 65 year age group to combine these findings. We measured heterogeneity across studies using the *I* ^*2*^ statistic, with higher values reflecting increasing heterogeneity. We assessed sources of heterogeneity by subgroup analysis and publication bias by examining funnel plots. Stata version 12.1 (StataCorp, College Station, TX) was used for analysis. Statistical tests were two sided with *p* < 0.05 defining statistical significance.

## Results

### Search results and study characteristics

**Fig. 1:**
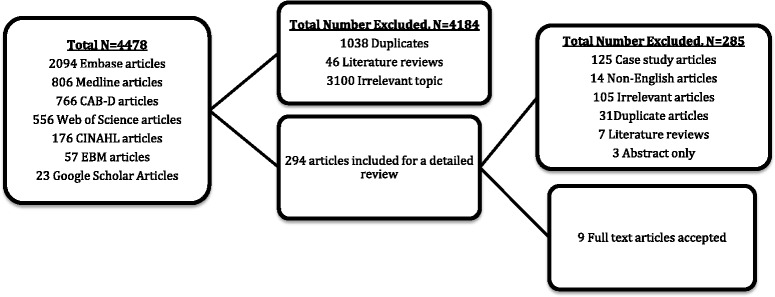
Study selection for inclusion into the meta-analysis

Figure 1 identifies the search results and selection process. Of the 4478 articles identified through the database search 1038 were removed due to duplicates and 46 articles were reviews of literature with topics such as pathophysiology and clinical features as well as treatment of herpes zoster, and 3100 articles were considered as irrelevant leaving 294 articles which were reviewed in detail by two individuals. Articles were deemed irrelevant if they didn’t meet the inclusion criteria, i.e. examined varicella or chickenpox instead of shingles, examined encephalitis instead of stroke with zoster, examined children primarily, examined herpes simplex instead of varicella zoster, examined vaccination, considered irrelevant outcomes (i.e. biomarker studies, cognitive impairment, stress). Of the 294 articles considered for a detailed review, the majority considered an irrelevant topic, while some were cases studies, non-English, or duplicates. This left 12 studies for inclusion in the review, nine studies with a full published manuscript [14–22] and 3 abstracts. Since the necessary results from the abstracts were unavailable and investigators declined to provide us with these when contacted, all 3 abstracts were excluded from analysis, leaving 9 studies for inclusion in our meta-analysis.

The characteristics of the 9 studies included in the meta-analysis are displayed in Table 1. Two of the earliest studies used the Taiwanese National Health Insurance Research Database (NHIRD) [15, 17]. Both Breur et al. [14] and Langan et al. [16] used the United Kingdom’s Clinical Practice Research Datalink (CPRD) and The Health Improvement Network (THIN) general practice databases respectively. The European studies conducted by Sreenivasan et al. [21] and Sundstrom et al. [19] used the Danish and Swedish registries, respectively. The two USA-based studies used Medicare [18] and Olmsted County residents, [20] respectively and the latest study used the Korean Health Insurance database [22]. Two studies performed a self-controlled case series analyses, [16, 18] while the other seven are retrospective cohort in design [14, 15, 17, 19–22]. Follow-up time varied from 1 to 24 years, but most studies reported the risk over various time periods post zoster infection.

**Table 1:** Characteristics of included studies

	Breuer	Kang	Kwon	Langan	Lin	Minassian	Sreenivasan	Sundström	Yawn
Year	2014	2009	2016	2014	2010	2015	2013	2015	2016
Design	Retrospective Matched Cohort	Retrospective Matched Cohort	Retrospective Matched Cohort	Self-Controlled Case Series	Retrospective Matched Cohort	Self-Controlled Case Series	Retrospective Cohort	Retrospective Cohort	Retrospective Matched Cohort
Data Source, Country	The Health Improvement Network (THIN) general practice database, United Kingdom	Taiwan National Health Insurance Research Database (NHIRD), Taiwan	Korea Health Insurance Database, Korea	UK Clinical Practice Research Datalink (CPRD) general practice database, United Kingdom	Taiwan National Health Insurance Research Database (NHIRD), Taiwan	Medicare administrative data claims, USA	Danish Civil Registration System (CRS), Denmark	Västra Götaland County Primary Health Care Register and the Swedish Patient Register, Sweden	Rochester Epidemiology Project, USA
HZ diagnosis period	2002–2010	1997–2001	2003–2013	1987–2012	2003–2004	2006–2011	1995–2008	2008–2010	1986–2011
Number of Cases (HZ and HZO)	106,601	7,760	70,424	6,584	658	42,954	117,926	13,296	4,478
Number of Controls	213,202	23,280	695,755	0	1,974	0	4,503,054	~1,500,000	16,800
Follow-Up Period	24 years	1 year	11 years	1 year	1 year	1 year	14 years	1 year	3 years
Gender, % female	59	52	49	57	51	71	51	60	62
Mean age at zoster diagnosis, years	59	47	41	Median age at stroke onset 77 years	57	80	Not reported	59	68
% Receiving AV Therapy	Not reported	Not reported	Not reported	55%	24% of cases	100%	50%	Not reported	Not reported
Case Inclusion Criteria	Adults 18 years of age or older diagnosed with incident HZ or HZO (index date as recorded in database)	Adults 18 years of age or older presenting to ambulatory clinic with incident HZ or HZO (index date as recorded in database)	Adults 18 years of age or older with incident HZ	Adults 18 years of age or older with incident HZ or HZO and incident stroke	Adults 18 years of age or older presenting to ambulatory clinic with incident HZO (index date as recorded in database)	Adults 65 years of age or older with evidence of incident HZ or HZO and an incident ischemic/nonspecific stroke	Adults 18 years of age or older who received acyclovir 800 mg in packs of 35	All individuals recorded in either of the two databases with incident HZ	Adults 50 years and older with incident HZ
Case Exclusion Criteria	Patients who experienced cardiovascular/stroke event (MI, TIA, stroke) before index date and those with recurrent HZ	Patients who had been diagnosed with stroke before the index date	Patients who had been diagnosed with stroke before HZ	Patients with evidence of HZ, postherpetic neuralgia, or stroke before the study period. Patients with incident episodes of TIA and subarachnoid hemorrhage or risk factors for subarachnoid hemorrhage. Patients with encephalitis 12 months after stroke. Patients with nonspecific cerebral aneurysms.	Patients diagnosed with HZO during the previous 1-year period. Patients diagnosed with any type of stroke prior to index ambulatory care visit. Patients with systemic lupus erythematosus, rheumatoid arthritis, multiple sclerosis, HIV, malignancy, and use of steroids or immunosuppressants for more than 1 month within 1 year prior to index date.	Individuals with evidence of vascular events or HZ before observation period. Secondary inpatient diagnosis of zoster or cardiovascular events. Individuals with subarachnoid hemorrhage (or established risk factors of) or encephalitis diagnosed up to 12 months after stroke	Patients who received a second acyclovir prescription of same strength and pack size. Patients with a stroke or TIA diagnoses before the start of the follow up period as per the ICD10 codes below as well as ICD8 codes 430-438	Patients diagnosed with HZ during the previous 1-year	Recurrent herpes simplex infection, history of stroke great than 1 month before index date, MI before index date
HZ Definition	Read codes corresponding to diagnoses of incident HZ (index date as recorded in database)	Incident HZ diagnosis using ICD9 codes 053x (index date as recorded in database)	ICD10 codes corresponding to diagnoses of incident HZ (index date as recorded in database)	Read or ICD10 codes corresponding to diagnoses of incident HZ	N/A	HZ diagnosis using ICD-9 codes 053x AND antiviral therapy 7 days before or after diagnosis	Patients who filled a single prescription of acyclovir 800 mg for 35 tablets	Incident HZ diagnosis using ICD-10 code B02.X	Incident HZ diagnosis using ICD-9 codes, and confirmed by medical record review
HZO Definition	Read codes corresponding to diagnoses of incident HZO	Incident HZO diagnosis using ICD9 code 053	N/A	Read codes corresponding to diagnoses of incident HZO	Incident HZO diagnosis based on the ICD9 code 053.2	HZO diagnosis using ICD-9 codes AND antiviral therapy 7 days before or after diagnosis	N/A	N/A	N/A
Stroke Definition	Read codes corresponding to stroke, diagnosis	Stroke/TIA diagnosis as per ICD9 codes 430-438	Stroke/TIA diagnosis as per ICD10 codes	Read or ICD10 codes corresponding to stroke diagnosis	Stroke/TIA diagnosis as per ICD9 codes 430-438	Ischemic/nonspecific stroke diagnosis as per ICD9 codes 436, 433x1, or 434x1	Diagnosis in hospital of stroke/TIA as per ICD10 I60-64 and G45	Stroke diagnosis as per ICD-10 codes I61-I64 (excluding I62)	Stroke diagnosis using ICD-9 codes
Control selection	Patients who had no record of HZ, matched (2:1) by age (+/−2 years), sex, and general practice	Patients with no HZ or stroke before 2001, matched (3:1) on age and sex, and defined their index date as their first ambulatory care visit in 2001	Patients without HZ, matched on age group	Self controlled	Selected from remaining patients, matched (3:1) on age group and gender, and defined their index date as their first ambulatory care visit in 2004	Self controlled	Patients who had no prior history of acyclovir, valacyclovir, or famciclovir use (as a proxy for unexposed)	Total remaining population without HZ	Matching each patient with HZ with up to 4 patients whose birthday was +/− 1 year, who were the same sex, and no HZ in the past 5 years
Confounders (Adjusted for)	Age, sex, obesity, smoking, high cholesterol recording, hypertension, diabetes, ischemic heart disease, atrial fibrillation, intermittent arterial claudication, carotid stenosis, and valvular heart disease	Age, sex, hypertension, diabetes, coronary heart disease, hyperlipidemia, renal disease, atrial fibrillation, heart failure, heart valve/myocardium disease, carotid/peripheral vascular disease, monthly income, urbanization level, and geographical region	Age, male gender, hypertension, hyperlipidaemia, ischaemic heart disease, diabetes, heart failure, peripheral vascular disease, arterial fibrillation or atrial flutter, renal disease and valvular heart disease	Confounders are implicitly controlled for due to study design	Age, sex, hypertension, diabetes, hyperlipidemia, coronary heart disease, chronic rheumatic heart disease, other forms of heart disease, and medication habits	Confounders are implicitly controlled for due to study design	Age, sex, calendar period, acute MI, atrial fibrillation, education, cancer, medications (antihypertensives, drugs used to treat dyslipidemia and atrial fibrillation, immunosuppressive drugs)	Age and sex	Age, sex, hypertension, dyslipidemia, coronary artery disease (including MI), arrhythmias, congestive heart failure, diabetes, depression, chronic obstructive pulmonary disorder, vasculopathies, stroke, and anxiety
Relevant study outcomes	Stroke HR after >1 year follow up, with analyses stratified by age <40 and ≥40 years, and by HZO.	Stroke HR after 1 year follow up since HZ, with analyses stratified by age (<45 and ≥ 45 years), gender and by HZO	Stroke HR after 11 years of follow up, stratified by age 18–30, 30–40, 40–50, 50–60, 60–70, and >70 years	Stroke IR 1–4, 5–12, 13–26, and 27–52 weeks after HZ or HZO +/−head and neck involvement, with analyses stratified by those who received antiviral therapy and by HZO	Stroke HR after 1 year follow up since HZO, with analyses stratified by those who received antiviral therapy	Stroke IR at 1, 2–4, 5–12, 13–26, and 27–52 weeks since HZ diagnosis, with analyses stratified by gender, HZO	Stroke IRR after <2 weeks, 2–52 weeks, and >1 year follow up since HZ, with analyses stratified by age <40, 40–59, and ≥60 years and by gender	Stroke IRR after 1 year follow up, with analyses stratified by age <40, 40–49, 50–59, 60–69, 70–79, and ≥80 years and gender	Stroke OR 3 and 6 months and 1 and 3 years after zoster.

*HZ* herpes zoster, *HZO* herpes zoster ophthalmicus, *MI* myocardial infarction, *TIA* transient ischemic attack, *HIV* human immunodeficiency virus, *BMI* body mass index, *IQR* interquartile range, *ICD* International Classification of Diseases, *HR* hazard ratio, *IR* incidence ratio, *OR* odds ratio

All studies adjusted for age and sex. All studies except Sundström et al. [19] adjusted for major cardiovascular risk factors such as hypertension diabetes, congestive heart failure, dyslipidemia, ischemic heart disease, atrial fibrillation, intermittent arterial claudication, carotid stenosis, and valvular heart disease. Only the study by Breuer et al. [14] was able to adjust for smoking and obesity (also considered as cardiovascular risk factors).

However the two studies by Minassian [18] and Langan [16] also provided strong control for confounding by using a self-controlled case-series design which only makes within-person comparisons. Antiviral therapy varied between studies with two studies including cases that were all taking antivirals, [18, 21] while Langan [16] and Lin [17] reported that 55% and 24% of their patients receiving antiviral therapy respectively. The mean age of the participants in each study ranged from 47 to 80 years. Langan et al. [16], Minassian et al. [18], and Yawn et al. [20] included the oldest participants with mean ages of 77 (median), 69, and 80 years, respectively. The proportion of women was higher than men in most of the studies.

Four studies examined the combined outcome of stroke or TIA, [15, 17, 21, 22] whereas the other five studies examined the risk of stroke only [14, 16, 18–20]. All studies examined stroke risk following zoster except Lin et al. [17] who examined stroke risk after herpes zoster ophthalmicus only. An additional four studies also included an analysis of stroke risk following ophthalmicus [14–17, 22]. Sreenivasan et al. [21] used acyclovir prescriptions filled in a certain strength and quantity to identify cases of HZ but used ICD10 codes to identify incident stroke and TIA. The rest of the studies used diagnosis codes to identify their HZ cases and cerebrovascular events.

### Risk of bias

Assessment of study validity using the Newcastle-Ottawa Scale revealed a low risk of bias amongst studies (Additional file 1: Table S1). Among the nine studies three scored a full 9/9, five studies scored 8/9, and one study scored 6/9.

### Herpes zoster infection and risk of stroke

**Fig. 2:**
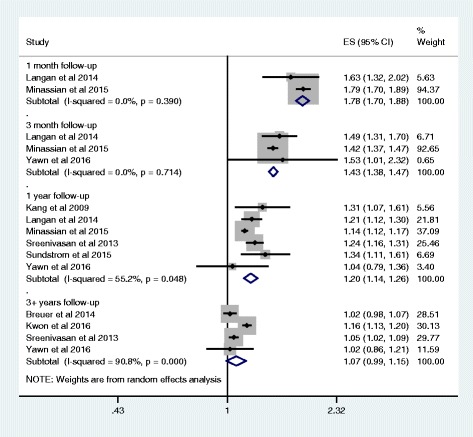
Effect of herpes zoster on stroke risk by length of study follow-up

Our meta-analysis indicates an elevated risk of stroke after zoster (Fig. 2). The risk of stroke was greatest during the first month following the HZ episode RR 1.78 (95% CI: 1.70-1.88 *I* ^*2*^ = 0%), dropping to 1.43 (95% CI: 1.38-1.47, *I* ^*2*^ = 0%) over the first 3 months following HZ, to 1.20 (95% CI: 1.14–1.26, *I* ^*2*^ = 55%) in the first year after the HZ episode, and finally to 1.07 (95% CI: 0.99–1.15, *I* ^*2*^ = 91%) over 3 or more years. For the studies with one year follow-up, the considerable heterogeneity was due to the smaller effect size reported by Minassian et al. [18]. This could be due to the self-controlled case-series design, the relatively older age of the patients and the specific outcome of ischemic stroke. For the studies with three or more years of follow-up, heterogeneity was introduced by the larger effect size reported by Kwon et al. [22]. This may reflect differences in the study population in the South Korean study or study design, such as misclassification of the TIA diagnosis. We did not find evidence of publication bias, however there were few studies.

**Fig. 3:**
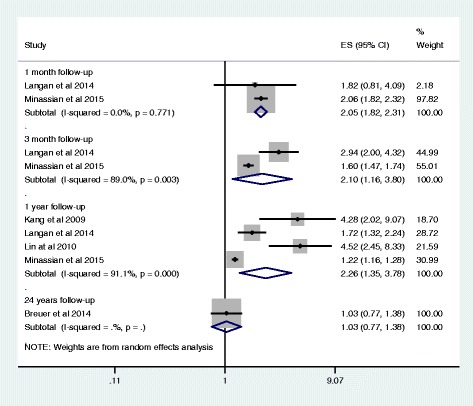
Effect of herpes zoster ophthalmicus on stroke risk by length of study follow-up

The risk of stroke following herpes zoster ophthalmicus appeared stronger compared to an episode of HZ only (1 month: RR 2.05 95% CI: 1.82-2.31, *I* ^*2*^ = 0%) as seen in Fig. 3. The risk also remains high in the first year after the HZ episode (RR 2.26, 95% CI: 1.35–3.78), but there was considerable heterogeneity (*I* ^*2*^ = 91%). This heterogeneity may be explained by the Langan et al. [16] and Minnassian et al. [18] studies using a self-controlled case series design, thus potentially having better confounding control than the other two cohort studies [15, 17]. Kang et al. [15] and Lin et al. [17] likely produced similar effect estimates as their data was ascertained from the same Taiwanese Health Insurance database, albeit in different patients over different time periods. Finally, the Breuer et al. [14] study found no excess risk over 24 years of follow-up.

**Fig. 4:**
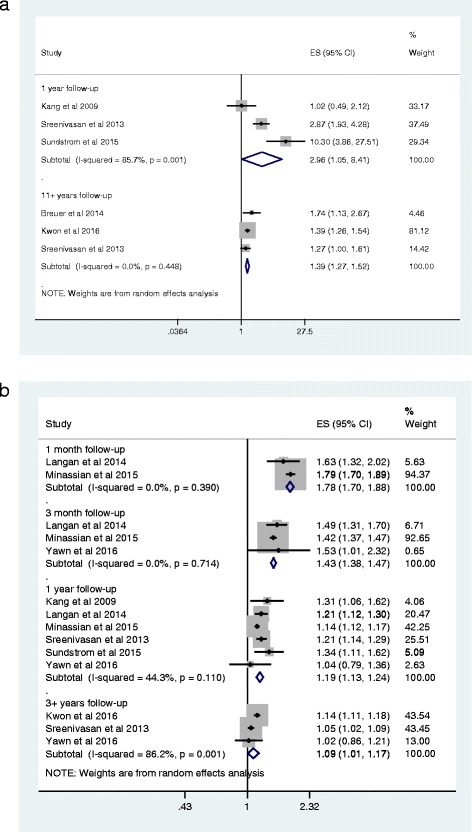
**a** Effect of herpes zoster on stroke risk by length of study follow-up and age (younger adults, aged 40 years or less). **b** Effect of herpes zoster on stroke risk by length of study follow-up and age (older adults, aged >40 years)

Figure 4 details stroke risk after HZ in different age groups. In younger adults (less than 40 years) there is a significant increased risk of stroke within a year after zoster episode (RR 2.96, 95% CI: 1.05-8.41, *I* ^*2*^ = 86%), but significant heterogeneity in these results. The larger effect size by Sundstrom et al. [19] could be due to not adjusting for any confounders beyond age and sex. The study cohort by Sreenivasan and colleagues [21] differed by being based on a treated population as they used use of antivirals as a proxy for identifying their HZ cases. For the younger aged cohort, the risk remains significant in the long-term, that is beyond 11 years after the acute HZ episode (RR 1.39, 95% CI: 1.27–1.52, *I* ^*2*^ = 0%). More studies evaluated stroke risk in older cohorts, and like the younger cohorts, we found that older adults also demonstrate a significant increase in stroke risk one year after zoster (RR 1.19, 95% CI: 1.13–1.24, *I* ^*2*^ = 44%), with this risk being greatest shortly after the acute zoster infection. The risk remains elevated beyond 3 years of follow up (RR 1.09, 95% CI: 1.01–1.17, *I* ^*2*^ = 86%), but with considerable heterogeneity in the results in line with the overall results where Kwon et al. [22] reported a greater effect size.

## Discussion

This is the first study to systematically review and perform a meta-analysis of the risk of stroke following a zoster infection. We found an elevated risk of stroke after pooling the results of nine observational studies. The risk was greatest shortly after the acute zoster episode but diminished slowly over time, although the risk was still significant after the first year. The risk of stroke was more pronounced in patients with herpes zoster ophthalmicus infection.

Varicella zoster virus is a highly neurotropic DNA virus that infects more than 95% of the world population [1]. Varicella infection or chickenpox most commonly occurs in children after which viral latency is established whereby the virus resides, but remains dormant, in the cranial nerve, dorsal root, and autonomic ganglionic neurons. During latency, varicella zoster virus transcription is limited and without production of virions. The increased stroke risk after reactivation of the varicella zoster virus may be due to the vasculopathy characteristically caused by this pathogen [23, 24]. By looking at brain tissue of individuals who have died from varicella infection, investigators believe the virus migrates transaxonally from the trigeminal nerves to cranial vasculature and spreads transmurally through the tunica adventitia, media, and intima, causing inflammation and thickening of the intima, reducing the media, and damaging the inner elastic layer of the vessels [24]. The presence of varicella zoster virus in intracerebral arteries is seen shortly after the acute infection and as late as 10 months after, allowing for the possibility that the risk of stroke could be present for up to a year after the initial HZ infection [25, 26].

The second possible mechanism is related to inflammation associated with systemic infection that can create a state in which the blood is more prone to clotting. Release of cytokines such as TNF-alpha and interleukin-2 during inflammation or stress leads to endothelial dysfunction disruption of atheromatous plaques and hypercoagulability, all leading to acceleration of atherosclerosis [27, 28]. A number of studies have suggested microbes play some role in inflammation, thereby accelerating atherosclerosis [29]. For example, Wang et al. conducted a meta-analysis that showed Helicobacter pylori infection contributes to risk of ischemic stroke,[30] however a more recent analysis refuted this [31]. In their meta-analysis, Chen et al. found an increased risk of cerebrovascular disease with the presence of IgG for Chlamydia pneumonia [32]. In a self-controlled case-series of over 50,000 patients, Smeeth et al., demonstrated a relationship between recent respiratory tract infection and myocardial infection (IR 4.95, 95%CI: 4.43–5.53) as well as stroke (IR 3.19, 95%CI: 2.81–3.62) [33]. The risks for both events were highest in the first 3 days, then gradually decreased to baseline the weeks following the acute infection.

We found evidence that the risk of stroke was present in both younger and older individuals but was more pronounced in the younger patients. However, this analysis was limited by only four studies that had one year follow-up data in patients less than 40 years of age, [15, 19, 21] and the results were considerably heterogeneous. One reason for this discrepancy could be that Sundstrom et al. [19] did not adjust for any confounders beyond age and sex and given that comorbidies would play a role in zoster incidence in the younger population, their results should be interpreted with caution. Further, in the younger population many risk factors may not be recorded or reported by patients and therefore despite adjustment, residual confounding may still be present. We were able to pool six studies to evaluate stroke risk in older patients one year after the acute HZ episode, giving less heterogeneous results. Despite differences in study populations, the studies were consistent in the magnitude of the risk being around 20% higher within the first year post HZ.

We observed a greater than 2-fold increase in stroke risk in the first year following herpes zoster ophthalmicus or zoster with trigeminal nerve involvement which as with general HZ decreased over time. The increased risk was not surprising given that HZO arises when latent VZV infection of the trigeminal ganglion becomes reactivated and involves the ophthalmic division of the trigeminal nerve, giving rise to a number of head and neck-related complications, including stroke or TIA [9, 26].

The pathogenesis behind increased stroke risk could also lead to increased risk for a myocardial infarction (MI). In our meta-analysis we did not address this risk and only two studies examined this outcome. Using a retrospective cohort of 106,601 HZ cases and 213,202 controls matched on age, sex, and general practice from a UK general practice database, Breuer et al. examined the risks of MI [14]. They found an increased risk overall (HR 1.10), but more pronounced in those less than 40 years of age (HR 1.49). Yawn et al. evaluated MI risk in 4,454 individuals with herpes zoster and 16,740 individuals without herpes zoster, all with no history of prior MI before their index date [20]. They found a greater risk of MI at 3 months post HZ (HR 1.68), but this reduced over time to a non-significant findings at 3 years (HR = 1.17). We agree with the authors of the two studies, that there is need for more studies to be conducted using large datasets to further examine the causal association.

There were too few studies for us to be able to examine stroke risk according to gender however the three studies that provided their results stratified by sex, found no differences in risk for men or women [15, 19, 20]. Clinical trials show that antivirals hasten the resolution of lesions, reduce the formation of new lesions and viral shedding as long as they are taken within 72 h after the onset of the rash [1]. The greatest benefit for these agents are in those at greatest risk for complications such those aged 50 years or older, have moderate to severe rash and/or pain, involvement of the face or eye, or are immunocompromised due to medications or disease. Unfortunately, there were too few studies for us to be able to examine stroke risk according to receipt of antiviral therapy, however, this would be an important piece of information as provision of antiviral therapy by clinicians may be of use for those adults with a high cardiovascular risk after zoster infection; further research is warranted on this topic. Similarly only one study examined the effect of vaccination on stroke risk after zoster, but had insufficient numbers of patients vaccinated in order to demonstrate a significant difference in stroke risk [18]. However, other studies have demonstrated that vaccination does reduce HZ incidence, hence they should be recommended for individuals at high vascular risk or with significant comorbidities [34]. Our findings focus solely on results from observational studies, although we acknowledge that randomized controlled trials would not be ethically possible in this setting. Observational studies are limited by their lack of randomization and inherent risk of bias. However, all nine studies were rated as having a low risk of bias and adjusted for important confounders such as demographics and a range of cardiovascular risk factors. Two studies implemented a self-controlled case series design, which potentially gives even stronger confounding control [16, 18]. However, we cannot rule out the possibility of some residual confounding due to stress, mental health or life events. The study by Sundström [19] had a greater risk of bias due to its primary aim being to assess HZ incidence, hence the recording of methods used to assess stroke risk were limited.

Our study is not without limitations. Occasionally we observed heterogeneity amongst studies results potentially due to different study designs, as previously mentioned. All of the studies except Yawn et al. [20], relied on electronic medical records to ascertain both the HZ and the stroke outcome. Administrative data has been reported to overestimate herpes zoster by 10–15%, [35] but other studies did make attempts to reduce HZ miscoding, by for example, excluding recurrent HZ, which may be confused with herpes simplex [14] or requiring concurrent antiviral therapy [18]. Regarding the accuracy of stroke recording, in a study of the UK databases nearly 90% of patients with stroke had diagnoses confirmed in their written medical record [36]. Studies varied as to whether their stroke definition included TIA or not, and those including TIA would have potentially overestimated risk as TIA is more common than stroke [14]. 10 or READ/10 or READ codes to identify zoster cases, study outcomes and confounders, except Yawn et al., [20] who used medical records, and Sreenivasan et al., [21] who used antivirals as a proxy of zoster infection. Given that the use of antivirals as surrogate for zoster infection has not been validated, we performed a sensitivity analysis and excluded this study from our analyses; however it did not change our results.

## Conclusions

Stroke risk is significantly increased shortly after acute zoster infection and remains elevated for up to one year. Herpes zoster ophthalmicus increases stroke risk by a larger magnitude. Increased efforts should be made to provide vaccinations to at-risk individuals, especially those with a high cardiovascular risk.

## Additional file

Additional file 1: Assessment for Risk of Bias. Risk of Bias Assessment for Cohort Studies Included in the Meta-Analyses Using the Newcastle-Ottawa Quality Assessment Scale. (DOCX 21 kb)

## Abbreviations

HZ: Herpes zoster
HZO: Herpes zoster ophthalmicus
PHN: Post-herpetic neuralgia
TIA: Transient ischemic attack
VZV: Varicella zoster virus
