# Supplementary material for: A meta-analysis of stroke risk following herpes zoster infection
Source: BMC Infect Dis. 2017 Mar 7;17:198. doi: 10.1186/s12879-017-2278-z (PMC5341420; doi:10.1186/s12879-017-2278-z)
Supplement: Additional file 1: — Assessment for Risk of Bias. Risk of Bias Assessment for Cohort Studies Included in the Meta-Analyses Using the Newcastle-Ottawa Quality Assessment Scale. (DOCX 21 kb) [file 12879_2017_2278_MOESM1_ESM.docx]

**Table e1:** Risk of Bias Assessment for Cohort Studies Included in the Meta-Analyses Using the Newcastle-Ottawa Quality Assessment Scale

| Study | Represen-tativeness of exposed cohort^1^ | Non- exposed cohort selection^2^ | Ascertain-ment of exposure^3^ | Outcome not present at start^4^ | Comparability^5^ | Outcome assessment^6^ | Follow-up long enough?^7^ | Adequacy of follow up^8^ | Number of stars |
| --- | --- | --- | --- | --- | --- | --- | --- | --- | --- |
| 1. Breuer  2014 | A* | A* | A* | A* | A*, B* | B* | A* | B* | 9 |
| 2. Kang  2009 | A* | A* | A* | A* | A*, B* | B* | B | B* | 8 |
| 3. Kwon  2016 | A* | A* | A* | A* | A*, B* | B* | A* | B* | 9 |
| 4. Langan  2014 | A* | A* | A* | A* | A*, B* | B* | B | B* | 8 |
| 5. Lin  2010 | A* | A* | A* | A* | A*, B* | B* | B | B* | 8 |
| 6. Sreenivasan  2013 | A* | A* | A* | A* | A* | B* | A* | B* | 8 |
| 7. Sundström 2015 | A* | A* | A* | B | A* | B* | B | B* | 6 |
| 8. Minassian 2015 | B* | A* | A* | A* | A*, B* | B* | B | B* | 8 |
| 9. Yawn  2016 | A* | A* | A* | A* | A*, B* | B* | A* | B* | 9 |

^1^ Cohort Representiveness: A* = truly representative of the average patients in the community (or patients with stroke in the community for the self-controlled case-series studies); B* = somewhat representative of the average patients in the community; C = selected group of users e.g. nurses, volunteers

^2^ Cohort Selection: A* = drawn from the same community as the exposed cohort; B = drawn from a different source

^3^ Exposure Ascertainment: A* = secure record (e.g. surgical records); B* = structured interview

^4^ Outcome: A* = Yes; B = No/unclear

^5^ Comparability: A* = study controls for age and sex B* = study controls for hypertension, hyperlipidaemia, diabetes

^6^ Outcome Assessment: A* = independent blind assessment; B* = record linkage; C = self-report

^7^ Follow-up greater than 1 year: A* = Yes, B = No

^8^ Adequacy of Follow-up: A* = complete follow up - all subjects accounted for; B* = subjects lost to follow up unlikely to introduce bias with a small number lost and a follow-up rate of greater than 80%; C = follow up rate less than 80% and no description of those lost
